# Editorial: Identification and characterization of novel antigens of malarial parasites, volume II

**DOI:** 10.3389/fcimb.2024.1408366

**Published:** 2024-04-15

**Authors:** Jin-Hee Han, Feng Lu, Yang Cheng, Md. Atique Ahmed

**Affiliations:** ^1^ Department of Medical Environmental Biology and Tropical Medicine, School of Medicine, Kangwon National University, Chuncheon, Republic of Korea; ^2^ Department of Pathogen Biology and Immunology, School of Medicine, Yangzhou University, Yangzhou, China; ^3^ Department of Public Health and Preventive Medicine, Wuxi School of Medicine, Jiangnan University, Wuxi, China; ^4^ Malaria Division, Indian Council of Medical Research (ICMR)-Regional Medical Research Centre, Dibrugarh, Assam, India

**Keywords:** malaria, Plasmodium, protein, antigen, antibody, Plasmodium falciparum, Plasmodium vivax, Plasmodium knowlesi

Malaria, a persistent global health challenge, continues to demand innovative solutions as we strive towards eradication. In this editorial volume II, we explored recent advancements in functional characterization of novel antigens in our understanding of Plasmodium biology both in human (*Plasmodium* vivax and *Plasmodium falciparum*) and non-human primate species i.e. *Plasmodium knowlesi* as revealed by a collection of insightful articles contributed to this Research Topic for future vaccine development and anti-malarial drugs.

The original article written by Won et al. “*Functional Characterization of Plasmodium vivax Hexose Transporter 1*” sheds light on the functional characterization of *Plasmodium vivax* hexose transporter 1 (PvHT1), a crucial player in glucose uptake. The article focuses on understanding the glucose uptake properties of the *Plasmodium vivax* hexose transporter 1 (PvHT1), a crucial component for the parasite’s survival. Despite being widely distributed and challenging to eradicate due to drug resistance and dormant liver forms, *P. vivax* lacks comprehensive research on PvHT1. Researchers studied the PvHT1NK strain, expressing it in Xenopus laevis oocytes. They found that PvHT1NK mediates the transport of glucose in a time-dependent manner without sodium dependency. Comparative analysis with *P. falciparum* HT1 revealed conserved glucose binding properties, suggesting similarities in glucose uptake mechanisms between the two species. These findings support the development of novel anti-malarial drugs targeting PvHT1, offering potential strategies for starving the parasite and combating vivax malaria. The original article written by Tebeje et al. “*Naturally acquired antibodies to gametocyte antigens are associated with reduced transmission of Plasmodium vivax gametocytes to Anopheles arabiensis*” delves into the intricate interplay between naturally acquired antibodies and malaria transmission. By investigating associations between antibody prevalence and P. vivax infectivity to mosquitoes, the study uncovered potential avenues for reducing transmission rates. Antibodies against specific gametocyte antigens (Pvs47, Pvs230 and Pvs25) demonstrated promising reductions in mosquito infection rates, offering hope for the development of transmission-blocking interventions and vaccine strategies. A similar original article written by Rahim et al. “*Naturally acquired antibody response to Plasmodium falciparum and Plasmodium vivax among indigenous Orang Asli communities in Peninsular Malaysia*” aimed to assess malaria exposure levels among indigenous Orang Asli communities in Kelantan, Peninsular Malaysia. The survey conducted from June to July 2019 revealed that overall malaria seroprevalence was 38.8% for PfAMA-1, 36.4% for PfMSP-119, 2.2% for PvAMA-1, and 9.3% for PvMSP-119. The study found a higher level of P. falciparum transmission compared to P. vivax. Living in certain areas, particularly Pos Kuala Betis, was associated with higher seropositivity rates for both P. falciparum and P. vivax. Age was also significantly associated with seropositivity to malaria antigens. The findings highlight the importance of serological data in understanding malaria transmission dynamics and guiding surveillance efforts in low-transmission settings like Peninsular Malaysia.

The original article written by Iyamu et al. “ *A conserved epitope in VAR2CSA is targeted by a cross-reactive antibody originating from Plasmodium vivax Duffy binding protein*” investigates the cross-reactivity of antibodies to VAR2CSA, a protein associated with placental malaria caused by Plasmodium falciparum, with antibodies raised against P. vivax Duffy binding protein (PvDBP). The researchers found that antibodies to PvDBP can cross-react with VAR2CSA, potentially generated by P. vivax infection in non-pregnant individuals. They identified the epitopes targeted by a specific monoclonal antibody raised against PvDBP and designed a synthetic peptide (CRP1) based on these epitopes. CRP1 was found to bind directly to chondroitin sulfate A (CSA), a receptor involved in placental sequestration. Antibodies raised against CRP1 significantly blocked the binding of infected erythrocytes to CSA *in vitro*. Seroreactivity to CRP1 was observed in at least 45% of Colombian cohorts, and antibody reactivities to CRP1 correlated strongly with those to the natural epitope in PvDBP. These findings suggest CRP1 as a potential vaccine candidate to target a distinct CSA binding site in VAR2CSA.

The last article by Ito et al. “*Roles of the RON3 C-terminal fragment in erythrocyte invasion and blood-stage parasite proliferation in Plasmodium falciparum*” focuses on the role of the protein RON3 in Plasmodium falciparum, the parasite responsible for malaria. RON3 plays a crucial part in parasite invasion of red blood cells and the subsequent development within them. By investigating different fragments of RON3, the researchers found that the C-terminal fragment is vital for parasite survival during the blood stage. Knockdown of this fragment led to defects in invasion and subsequent parasite growth. Additionally, they observed that the C-terminal fragment is involved in protein export and glucose uptake by the parasite. However, the C-terminal fragment’s role seems to be specific to the late ring stage of parasite development. This study sheds light on the intricate mechanisms involved in malaria parasite biology, particularly in erythrocyte invasion and nutrient acquisition. Article 5 provides deeper insights into the mechanisms underlying erythrocyte invasion by Plasmodium species, focusing on the role of the RON3 C-terminal fragment. By elucidating the essential functions of RON3 in blood-stage parasite survival and progression, the study enhances our understanding of parasite biology and identifies potential targets for intervention.

Collectively, the articles presented in this Research Topic offer a multifaceted exploration of Plasmodium vivax and Plasmodium falciparum, spanning from molecular mechanisms to epidemiological insights. These findings not only contribute to knowledge in malaria research but also pave the way for novel interventions, therapeutic strategies, and vaccine development.

## Author contributions

J-HH: Writing – review & editing. FL: Writing – review & editing. YC: Writing – review & editing. MA: Writing – original draft, Writing – review & editing.

